# Task-specific fear influences abnormal trunk motor coordination in workers with chronic low back pain: a relative phase angle analysis of object-lifting

**DOI:** 10.1186/s12891-022-05118-x

**Published:** 2022-02-18

**Authors:** Ren Fujii, Ryota Imai, Hayato Shigetoh, Shinichiro Tanaka, Shu Morioka

**Affiliations:** 1 Department of Neurorehabilitation, Graduate School of Health Science, Kio University, 4-2-2 Umaminaka, Koryo-cho, Kitakatsuragi-gun, Nara, 635-0832 Japan; 2 Department of Rehabilitation, Medical Corporation Tanakakai, Musashigaoka Hospital, 7-15-1 Kusunoki, Kita-ku, Kumamoto-shi, Kumamoto, 861-8003 Japan; 3 School of Rehabilitation Osaka Kawasaki Rehabilitation University, 158 Mizuma, Kaizuka-shi, Osaka, 597-0104 Japan; 4 Department of Physical Therapy, Faculty of Health Sciences, Kyoto Tachibana University, 34 Ooyakeyamada, Yamashina-ku, Kyoto-shi, Kyoto, 607-8175 Japan; 5 Neurorehabilitation Research Center, Kio University, 4-2-2 Umaminaka, Koryo-cho, Kitakatsuragi-gun, Nara, 635-0832 Japan; 6 Department of Rehabilitation Medicine, Medical Corporation Tanakakai, Musashigaoka Hospital, 7-15-1 Kusunoki, Kita-ku, Kumamoto-shi, Kumamoto, 861-8003 Japan

**Keywords:** Low back pain, Work-related activity, Lifting, A relative phase angle analysis, Mean absolute relative phase, Deviation phase, Kinematic

## Abstract

**Background:**

Pain-related fear influences impaired trunk movement (e.g., limited movement of range and velocity), but it is unclear how fear relates to trunk motor coordination (e.g., a more “in-phase” upper-lower trunk motion pattern). We conducted the present study to: (1) identify the motor coordination pattern of the in-phase upper-lower lumbar movements during the lifting, and (2) determine how pain-related fear is related to the trunk coordination pattern in workers with chronic low back pain (CLBP).

**Methods:**

We examined 31 male workers with CLBP (CLBP group) and 20 healthy controls with no history of CLBP (HC group). The movement task was lifting a box, the weight of which was 10, 30%, or 50% of the subject’s body weight. We used a 3D motion capture system to calculate the mean absolute relative phase angle (MARP) angle as an index of coordination and the mean deviation phase (DP) as an index of variability. We used a numerical rating scale to assess the subjects’ task-specific fear.

**Results:**

The MARP angle during trunk extension movement in the 50% condition was significantly decreased in the CLBP group compared to the HCs; i.e., the upper lumbar movement was more in-phase with the lower lumbar movement. The hierarchical multiple regression analysis results demonstrated that a decreased MARP angle was associated with high task-specific fear.

**Conclusions:**

A more ‘in-phase’ upper-lower lumbar movement pattern was predicted by task-specific fear evoked when performing a work-related activity. Our findings suggest that an intervention for task-specific fear may be necessary to improve an individual’s impaired trunk motor coordination.

## Background

Low back pain (LBP) is an important health problem in the work environment [1]. The estimated lifetime prevalence of LBP among workers is 63–83% [2, 3], and the condition of many individuals with LBP progresses to chronic LBP (CLBP) [3]. Low back pain impairs an individual’s trunk movement, which can lead to work-related disability, decreased work productivity, and decreased quality of life [4–6]. It has been demonstrated that impaired trunk movement due to LBP limits an individual’s movement range and velocity and is associated with psychological factors in addition to pain intensity of the LBP [7, 8]. Thomas et al. reported that smaller and slower trunk movements are consistently observed in patients with acute LBP with high fear of movement [8]. However, it is unclear whether mild LBP influences trunk movement, and even in people with recurrent LBP in remission, there seems to be a difference in movement coordination [9].

The impaired trunk movements in LBP can thus be characterized based on continuous data (such as upper-lower lumbar and trunk-lower limb movement coordination during movement) rather than on discrete data (e.g., limits in the segment range of motion and angular velocity) [9]. A systematic review reported that there was a significant difference in the trunk’s maximum range of motion between healthy individuals and those with LBP [10]. However, one certain kinematic study reported that no differences in trunk kinematics were observed, although there is variability in the symptoms of LBP [11]. Another study observed that during a lifting task, the subjects with LBP exhibited trunk, hip, and knee movements that coincided spatiotemporally, which is called the “in-phase coordination pattern” [12]. Among the various coordination patterns, the in-phase upper-lower lumbar coordination pattern during lifting directly influences the lower back’s load, which could lead to an exacerbation of lower-back injury [13].

It has been contended that such movement disorders (i.e., limited movement range and velocity) help the human body avoid incurring a back injury [7, 8]. Meier et al. indicated that protective behavior might be beneficial in cases of acute-phase injury, but it becomes maladaptive in cases of chronic-phase injury [14]. According to the fear-avoidance model of pain, misinterpretations of pain as being harmful may give rise to pain-related fear, resulting in protective behavior that is intended to avert bodily threat [15]. It appears that impaired trunk movement is also caused by pain-related fear in addition to sensory pain [7, 8].

Several kinematic studies reported that subjects’ impaired trunk range of motion and impaired velocity were affected by pain-related fear as measured by the Tampa Scale for Kinesiophobia (TSK), which is an index of general pain-related fear [16, 17]. Thus, trunk movement impaired by LBP is likely to be protective behavior caused by pain-related fear. The in-phase upper-lower lumbar coordination pattern during lifting, which has been considered merely an adaptive behavior in response to trunk instability and increased mechanical load [13], might be associated with limited freedom of trunk movement as avoidance behavior. Although quantitative kinematic analysis of trunk movement patterns has the potential to aid clinical assessments [18], the question of whether the in-phase coordination pattern represents avoidance behavior has not been addressed. In addition, although the TSK is frequently used to assess pain-related fear, there might be a difference between activities daily of life (ADLs) and work-related activity regarding the arousal of fear. Individuals with CLBP who were threatened only by specific movements despite having low TSK scores have also been described [19].

We conducted the present study of workers with CLBP to: (1) identify the motor coordination pattern of in-phase upper-lower lumbar movement during the lifting of an object, and (2) determine how pain-related fear is related to the trunk coordination pattern in workers with CLBP. We hypothesized that task-specific fear caused by lifting an object would result more often in the in-phase upper-lower lumbar motion coordination pattern.

## Subjects and methods

### Study design and subjects

The study subjects were nurses and caregivers who engaged in physical labor on hospital wards (i.e., patient transfer and carrying heavy objects). The recruitment period was from May 14, 2019, to August 21, 2019. Questionnaires and consent forms were distributed to the entire nurse and caregiver (*n* = 123) in the Musashigaoka Hospital. Nurses and caregivers who did not agree were excluded from the subject to carry out research based on the will of the individuals (*n* = 25). The study subjects were assigned to the CLBP group and healthy control (HC) group based on the inclusion criteria and exclusion criteria.

The definition of LBP was pain present from a lower rib edge to the gluteal fold [20]. The inclusion criteria were as follows: Male workers aged 20–40 years; LBP duration of > 3 months; and a score ≥ 1 on a numerical rating scale (NRS) for pain intensity during work in the past 4 weeks. Subjects were excluded if they had (1) a previous diagnosis of spinal disease (lumbar disc herniation, lumbar spondylolisthesis, or lumbar osteoarthritis), (2) pain in peripheral joints in an upper or lower limb, (3) the presence of neurological symptoms of a lower limb, (4) serious spinal pathology (cancer, inflammatory arthropathy, or acute vertebral fracture), or a diagnosis of neurological disease. The definition of HC had no history of LBP never before and no other diagnosis illnesses.

This study obtained ethical approval from the institutional ethics committee of Kio University (R2–01) and was conducted in compliance with the Declaration of Helsinki.

#### Procedure

Before the experimental task, the severity of the subjects’ LBP was assessed with the use of an NRS asking about the subject’s maximum pain intensity in the past 4 weeks, the TSK [21], the Pain Catastrophizing Scale (PCS) [22], the Fremantle Back Awareness Questionnaire (FreBAQ) [23], the Roland-Morris Disability Questionnaire (RDQ) [24], and Von Korff’s grading for the severity of LBP [25] as general measures of pain-related factors. The details of the questionnaires are as follows.

##### Pain assessment

The subjects’ pain intensity was assessed by an 11-point NRS (0 = no pain and 10 = highest possible degree of pain) to describe the subject’s maximum pain in the past 4 weeks (Pain NRS). The reason for focusing on maximum pain instead of average pain is that in our previous study, the subjects’ maximum pain intensity was indirectly associated with impaired trunk movement [26].

##### Pain-related psychological assessment

The subjects’ kinesiophobia was assessed by the 11-item Japanese version of the TSK (TSK-11) which shows better internal reliability, identical construction, and known group validity compared to the 17-item version [21]. This assessment was scored on a four-point scale from 1 (strongly disagree) to 4 (strongly agree), and the possible score ranges from 11 to 44; a higher score indicates a higher degree of pain-related fear [21]. For the assessment of catastrophic thinking, we used the four-item version of the PCS (PCS-4), a shorter version of the 13-item PCS [22]. This assessment was scored on a 5-point scale from 0 (not at all) to 4 (all the time), and the possible scores range from 0 to 16; a higher score indicates a higher degree of catastrophic thinking [22]. The PCS-4 was confirmed to have good internal reliability and internal consistency [22]. We used the TSK-11 and PCS-4 in this study because they have properties that are similar to those of the original scales but offer the advantages of brevity.

##### Body perception assessment

To assess the subjects’ body image of their lower back region, we used the FreBAQ [23], which is a nine-item questionnaire; it is based on a five-point response scale from 0 (never) to 4 (always), and the possible scores range from 0 to 36. A higher score indicates more disturbed perception.

##### Pain-related disability assessment

The RDQ was used to assess disability directly related to LBP [24]. This assessment is a 24-item questionnaire with a dichotomous scoring format; yes (= item is applicable), or no (= item is not applicable). A high score indicates a higher degree of LBP-related disability. For the evaluation of the severity of the subjects’ LBP, Von Korff’s grading was used as follows: grade 0 = no LBP; grade 1 = LBP that does not interfere with work; grade 2 = LBP that interferes with work but does not cause absences; and grade 3 = LBP that interferes with work, leading to sick leave [25].

After each subject’s assessment by the above-described questionnaires, a movement analysis was performed during a lifting task. We used a lifting task because it is a work-related activity that is widely recognized as a risk factor for LBP and has been used as an experimental task in kinematic studies [27]. After performing the lifting task, our subjects were also asked to complete task-specific questionnaires about pain, discomfort, pain expectation, and pain-related fear with the use of an NRS. These task-specific questionnaires were administered after each lifting condition.

#### Experimental task

**Fig. 1:**
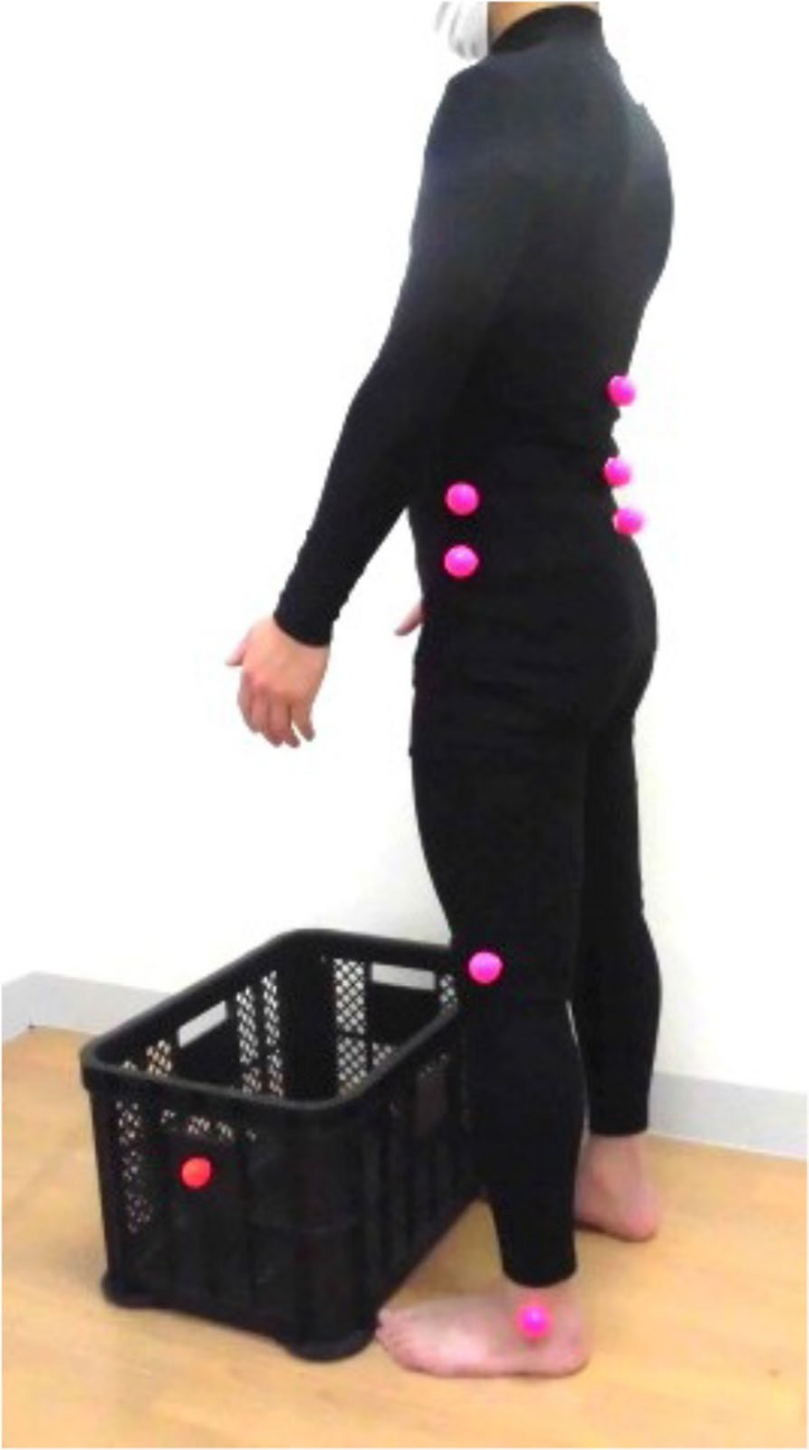
The subjects were asked to lift a box (520 × 365 × 305 mm) placed on the ground. The attachment positions of the 11 markers (30-mm dia.) are shown: the subject’s thoracic spine (Th12 spinous process), lumbar (L3 spinous process), pelvic (S1 spinous process), and bilaterally on the lilac crest, great trochanter, lateral femoral epicondyle, and lateral malleolus. A marker was also attached to the box

An experimental task that involved lifting an object was used. The subjects were asked to lift a box (520 × 365 × 305 mm) placed on the ground (Fig. 1). The subject’s start position was standing with the feet shoulder-width apart, and the centerline of the box width was placed to match the center of the subject’s feet. The box was placed so that there was no space between the subject’s toes and the box. The reason for these controlled factors is that an earlier study reported that the positional relationship between the subject’s feet and the box affected the low back load during lifting [28].

In the present study, the subjects were asked to initiate lifting the box as quickly as possible upon hearing the start cue, and to lift the box to waist-height. The weight of the box was 10, 30%, or 50% of the subject’s body weight. We used a block design in which the subjects first performed at the 10% of body weight condition and then the 30% of body weight condition, and finally the 50% of body weight condition. We used this design because another study of object-lifting reported that the weight of the object affected the lifting performance that follows [29]. After completing several practices with an unweighted box, our subjects performed the lifting task five times for each weight condition. They had a 1-min rest between trials.

### Instrumentation

We recorded trunk kinematic data during the lifting task by using a three-dimensional (3D) motion capture system with a four-charge-coupled device (CCD) camera (KinemaTracer, KisseiComtec, Matsumoto, Japan). The system recorded the displacement of color markers at a sampling frequency of 60 Hz. A total of 12 markers (30 mm in dia.) were attached to parts of the subject’s body and the box as shown in Fig. 1. Using palpation, a physical therapist with 10 years of experience identified the appropriate anatomical landmarks by using the technique suggested in *Gray anatomy for students* [30] and attached 11 markers to the subject: the thoracic spine (Th12 spinous process), lumbar spine (L3 spinous process), pelvis (S1 spinous process), bilaterally on the iliac crest, great trochanter, lateral femoral epicondyle, and lateral malleolus. The 12th marker was placed on the box.

### Coordination analysis

The recorded kinematic data obtained by the 3D motion capture system were low-pass filtered with a second-order recursive Butterworth filter with a cutoff frequency of 6 Hz. To define an upper lumbar angle, a vector was created based on the color markers placed on the Th12 spinous process and the L3 spinous process. A lower lumbar angle was defined as a vector-based on the color markers placed on the L3 spinous process and S1 spinous process. The Upper and lower lumbar angles were calculated using the angle between each of these vectors and the vertical axis.

**Fig. 2:**
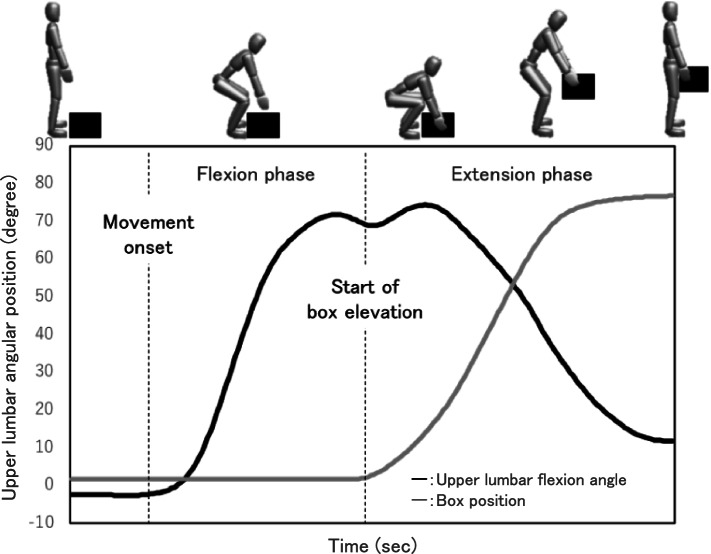
Time series variation of the trunk movement during lifting. The time series was divided into two phases according to upper trunk movement and box position

We divided the time series of trunk movement into flexion and extension phases, and we calculated the trunk coordination pattern of each phase (Fig. 2) [31]. The flexion phase began with the start of trunk flexion motion and ended when the box was raised. The extension phase started when the box was raised and ended when the trunk had resumed an upright position. In accord with previous research, we conducted a relative phase angle analysis by the following procedures [12, 32]. The moment the box left the ground was identified by the vertical axis of the marker attached to the box. The upper and lower lumbar angular displacement and angular velocity data were time-normalized to 100%. Before the plotting of the phase diagram, the angular displacement and angular velocity data were normalized to − 1 to + 1 intervals using the following equation:

$$Normalized\ angle:\left(\left[ angle-\mathit{\min}\ angle\right]/\left[\mathit{\max}\ angle-\mathit{\min}\ angle\right]\right)\times 2-1$$

$$Normalized\ angular\ velocity: angular\ velocity/\mathit{\max}\ angular\ velocity$$

**Fig. 3:**
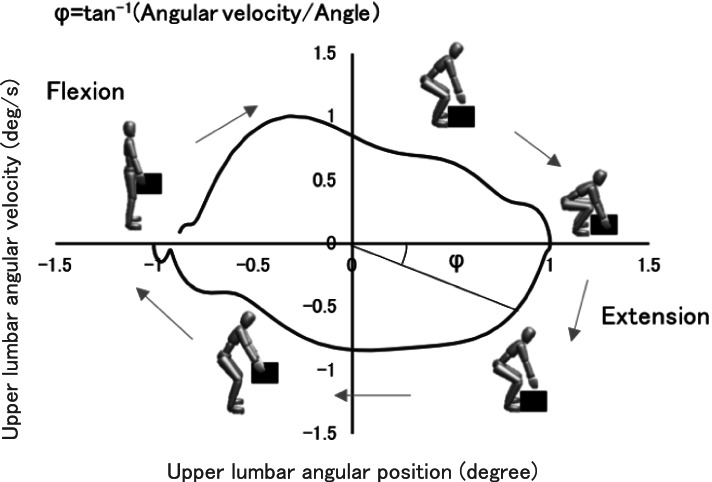
Phase diagram for the upper trunk segment. The phase angle, φ, at any point of flexion and extension can be calculated using the formula tan^−1^ (Angular velocity/angle). The phase angle was calculated in this study by dividing the flexion phase and extension phase

The normalized angular displacements were plotted versus the normalized angular velocity to generate the phase plane for each segment (Fig. 3). For the quantification of the phase plane trajectories, the phase angle was derived using the following equation:

$$\varPhi =\mathit{\tan}^{-1}\left( Normalized\ angular\ velocity/ Normalized\ angle\right)$$

For an index of upper-lower lumbar coupling, we calculated continuous relative phase (CRP) curves. The CRP was defined as the phase angle difference between the upper lumbar and the lower lumbar (i.e., φlower lumbar − φupper lumbar). To compare the CRP curves between the groups and each condition, we calculated the mean absolute relative phase (MARP) values for the upper and lower lumbar. A MARP angle closer to 0° indicates a more in-phase motion pattern between two segments, and a MARP angle closer to 180° suggests a more out-of-phase motion pattern. It was suggested that a more in-phase coordination pattern might indicate increasing protective behavior in the performance of a task [13]. Herein, the MARP was derived using the following equation:

$$MARP={\sum}_{i=1}^P \mid \kern0.50em \varphi CRP \mid i/P$$

To quantify the variability of coordination patterns, we calculated the deviation phase (DP). DP values closer to 0° indicate lower coordination variability or more coordination stability. The DP was derived using the following equation:

$$DP={\sum}_{i=1}^P\ SDi\kern0.5em /P$$

Each variable was averaged for each condition.

### Assessment of task-specific pain-related factors

To assess task-specific pain-related factors, we used an NRS (0 = no feeling and 10 = highest possible degree of feeling) that concerns the subject’s maximum pain, discomfort, pain expectation, and pain-related fear that occurred during the lifting task. All of the above assessments were conducted in each (10, 30, 50% body weight) lifting condition (i.e., three times). The subjects were asked to respond verbally to the NRS at the end (not the beginning) of each condition, based on a previous study [33]. We used these retrospective assessments because asking the subjects questions about their pain expectation and pain-related fear before they performed the lifting task might lead them to imagine a context other than the study’s task setting. The subjects were asked the following questions at the end of each (10, 30, 50%) condition: (1) “How much pain in your back did you feel when lifting the box?” (pain), (2) “How much discomfort in your back did you feel when lifting the box?” (discomfort), (3) “How much pain did you anticipate when lifting the box?” (pain expectation) and (4) “How much fear did you feel when lifting the box?” (pain-related fear).

It was reported that such task-specific measures of pain-related factors were more useful for the prediction of a limited lumbar range of motion in CLBP patients compared to general measures of pain-related pain (e.g., the TSK and PCS) [34].

#### Statistical analyses

The statistical analyses of the results were performed as follows. After using the Shapiro-Wilk test to confirm the normality of the basic information of the subjects (i.e., age, height, and weight), pain-related factors (i.e., the scores on the Pain NRS, TSK-11, PCS-4, RDQ, FreBAQ, and the task-specific measure of pain-related factors), and kinematic factors (the MARP and the DP), we compared these variables between the CLBP group and HC group using the Mann-Whitney U-test and Fisher’s exact test. To compare the difference in task-specific measures of pain-related factors and kinematic factors in each condition and group, we used a two-way repeated-measures analysis of variance (ANOVA). We performed a hierarchical multiple regression analysis to analyze the relationship between kinematic factors and pain-related factors. All statistical analyses were performed using HAD ver. 14.8 [35]. The level of significance for all analyses was set as *p* < 0.05.

1. *Mann-Whitney U-test and Fisher’s exact test*

We compared the subjects’ age, height, weight, LBP duration, and general measures of pain-related factors (Pain NRS, TSK-11, PCS-4, RDQ, and FreBAQ) between groups using the Mann-Whitney U-test. To compare the occupational category and LBP of severity, we used Fisher’s exact test.

2. *Two-way repeated measures ANOVA*

To compare the differences in task-specific measures of pain-related factors and kinematic factors in each condition and group, we used a two-way ANOVA. The binary factors were weight condition (10, 30, and 50% of body weight) and group (CLBP group and HC group). For post hoc comparisons, the Bonferroni method was used for multiple comparisons.

3. *Correlation analysis*

We used Spearman’s rank correlation coefficients to analyze the relationship between kinematic factors and task-specific measures of pain-related factors. In the correlation analyses, we focused on the variables in which there were significant interactions and main effects.

4. *Hierarchical multiple regression analysis*

A hierarchical multiple regression analysis was performed to test the hypothesis that pain-related fear contributes to the in-phase trunk motor pattern. We used a hierarchical multiple regression analysis because we needed to consider the effects of confounding factors, as impaired motor behavior in LBP patients involves complex interactions of various pain-related factors [14]. We thus performed the hierarchical multiple regression analysis with kinematic variables for which there was a significant interaction and main effect, plus demographic variables (age and LBP duration), general measures of pain-related factors, and the task-specific measurement of pain-related fear.

In model 1, age and LBP duration were the independent variables. In model 2, the general measures of pain-related factors (Pain NRS, TSK-11, PCS-4, FreBAQ) were added to model 1 as independent variables. In both models, we selected factors associated with impaired trunk movement in CLBP that had been reported as independent variables in an earlier study [17, 36, 37]. In model 3, the task-specific measurement of pain-related fear was added to model 2 as an independent variable.

## Results

**Fig. 4:**
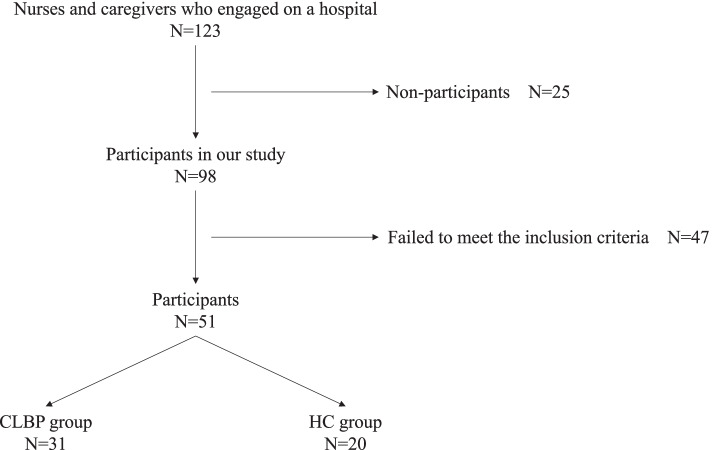
Flowchart of inclusion and exclusion criteria for this study

Figure 4 outlines the study’s recruitment of subjects. A total of 98 workers were screened as potential subjects; 47 subjects failed to meet the inclusion criteria. The final number of subjects for the analyses was thirty-one LBP group and 20 HC group. The power of sample size in this study was analyzed using a post-hoc analysis (G*Power 3.1), and the power was 0.70 (alpha level of 0.05, large effect size).

### Comparison of subject characteristics

Table 1 summarizes the subjects’ characteristics. There were no significant differences between the CLBP and HC groups in age, height, weight, or occupational category. Regarding the evaluation of pain-related indicators, the NRS, TSK-11, FreBAQ, and RDQ scores in the CLBP group were all significantly higher than those in the HC groups. Significant differences in the severity of LBP were revealed between the groups; there were 30 subjects at stage1 and five subjects at stage 2 in the CLBP group, and 20 subjects at stage 0 in the HC group. The PCS-4 scores showed no significant between-group difference. All subjects completed the experimental task.

**Table 1:** The subjects’ characteristics and clinical information

	HC ***n*** = 20	CLBP ***n*** = 31
Age, yrs	28.1 (5.2)	30.5 (6.0)
Male sex, n	20 (100)	31 (100)
Height, cm	172.0 (4.8)	172.7 (3.6)
Weight, kg	66.7 (9.0)	65.5 (4.2)
Occupational category:		
Nurse	11	18
Care worker	9	13
Duration, months	–	14.7 (14.9)
Pain NRS; pain intensity in the past 4 wks, 0–100*	0 (0)	3.6 (1.7)
TSK-11, 11–44*	19.1 (5.1)	21.9 (5.0)
PCS-4, 0–16	5.5 (4.7)	6.4 (3.9)
FreBAQ, 0–36*	2.7 (4.3)	7.5 (6.5)
RDQ, 0–24*	0 (0)	2.1 (1.4)
Severity of LBP, n*	Grade 0: 20 (100)	Grade 1: 29 (93.5) Grade 2: 2 (7.5)

Values are means (SD) or n (%)

*FreBAQ* Fremantle Back Awareness Questionnaire, *NRS* Numerical rating scale, *PCS-4* Pain Catastrophizing Scale-4, *RDQ* Roland-Morris disability questionnaire, *TSK-11* Tampa Scale for Kinesiophobia-11

*p < 0.05 between the healthy control (HC) and chronic lower back pain (CLBP) groups

### Between-group comparison of task-specific pain factors in each condition

Table 2 provides the results of the comparison of task-specific pain factors in each condition between the CLBP and HC groups. Pain, discomfort, pain expectation, and pain-related fear showed significant main effects (*p* < 0.01) and interactions (*p* < 0.01). A post hoc test showed that all variables in the CLBP group in the 30 and 50% conditions were significantly higher than those in the HC group.

**Table 2:** Comparison of task-related pain factors among groups in each condition

	CLBP (***n*** = 31)			HC (***n*** = 20)			Repeated measures ANOVA		
	10% condition	30% condition	50% condition	10% condition	30% condition	50% condition		F	***p***-value
Pain	0.10 (0.30)	0.65 (1.09)^#^	2.35 (2.25) ^†^	0 (0)	0 (0)	0 (0)	Interaction	12.59	< 0.01
							Weight	29.01	< 0.01
							Group	12.59	< 0.01
Discomfort	0.61 (1.07)	1.81 (1.55)^#^	3.39 (2.66) ^†^	0 (0)	0 (0)	0.55 (1.12)	Interaction	6.24	< 0.01
							Weight	46.43	< 0.01
							Group	14.30	< 0.01
Pain expect	0.65 (1.21)	2.06 (1.78)^#^	4.52 (2.63) ^†^	0 (0)	0.55 (0)	1.45 (1.62)	Interaction	6.24	< 0.01
							Weight	37.78	< 0.01
							Group	30.03	< 0.01
Fear	0.29 (0.96)	1.81 (2.02)^#^	4.03 (2.75) ^†^	0 (0)	0.3 (0.95)	0.4 (1.07)	Interaction	7.01	< 0.01
							Weight	24.87	< 0.01
							Group	11.41	< 0.01

Values are means (SD). 10% condition: the condition with 10% of body weight, 30% condition: with 30% of body weight, 50% condition: with 50% of body weight

^#^*p* < 0.006 between HC and CLBP groups at 30% condition

^†^*p* < 0.006 between HC and CLBP groups at 50% condition

### Between-group comparison of kinematic variables in each condition

The results of our between-group comparison of the MARP and DP in each condition are given in Table 3. The MARP in the extension phase showed significant main effects in weight condition and group factors (*p* < 0.01) and interactions (*p* < 0.01). A post hoc test showed that the MARP values in the CLBP group in the 50% condition was significantly lower than those in the HC group. The DP in the extension phase showed a significant main effect in the weight condition factor, but no significant main effect in the group factor or interaction. The MARP and DP in the flexion phase showed no significant main effect or interaction.

**Table 3:** Comparison of MARP and DP among groups in each condition

	CLBP (***n*** = 31)			HC (***n*** = 20)			Repeated measures ANOVA		
	10% condition	30% condition	50% condition	10% condition	30% condition	50% condition		F	***p***-value
MARP	15.97 (6.47)	15.83 (6.60)	14.70 (6.58)	16.05 (4.97)	16.64 (4.86)	16.08 (5.20)	Interaction	0.14	0.87
Flexion phase							Weight	0.25	0.78
							Group	0.56	0.46
MARP	15.61 (5.92)	18.52 (7.49)	18.60 (6.43)^#^	16.43 (4.89)	17.97 (6.16)	25.46 (6.09)	Interaction	4.56	< 0.05
Extension phase							Weight	10.86	< 0.01
							Group	4.97	< 0.05
DP	0.13 (0.03)	0.13 (0.04)	0.12 (0.04)	0.13 (0.03)	0.14 (0.03)	0.14 (0.03)	Interaction	0.78	0.46
Flexion phase							Weight	0.77	0.47
							Group	2.78	0.10
DP	0.13 (0.04)	0.15 (0.04)	0.15 (0.03)	0.13 (0.03)	0.15 (0.03)	0.16 (0.03)	Interaction	0.30	0.89
Extension phase							Weight	5.34	< 0.01
							Group	0.32	0.57

Values are mean (SD). Conditions are explained in the Table 2 footnote

^#^*p* < 0.006 between CLBP and HC groups

### Correlations between trunk coordination variables and task-related pain factors

The results of the correlation analysis between the MARP and task-related pain factors are as follows. We used each variable in the 50% condition for which there was a significant interaction and the main effect. The MARP was significantly correlated with the task-specific measurement of pain-related fear (*r* = − 0.70, *p* < 0.01), but not with pain (*r* = 0.12, *p* = 0.21), discomfort (*r* = − 0.07, *p* = 0.29), or pain expectation (*r* = 0.007, *p* = 0.32).

### Hierarchical multiple regression analysis results

The results of the hierarchical multiple regression analysis are summarized in Table 4. In model 1, age and LBP duration were not significantly associated with the MARP. In model 2 (with the addition of the general measures of pain-related factors), the TSK-11 score was significantly associated with the MARP. From the adjusted ΔR^2^ results, the model accounted for an additional 16% of the variance, but we detected no significance in the coefficient of determination. In model 3, the task-specific measure of pain-related fear was significantly associated with the MARP. From the adjusted ΔR^2^ results, the model accounted for an additional 26% of the variance, which we observed was significant in the coefficient of determination. In other words, task-specific fear explained an extra 26% relative to model 2. Based on the values of the variance inflation factor, we did not find evidence of multicollinearity.

**Table 4:** Hierarchical regression analysis identifying the factors associated with in-phase trunk coordination

Dependent variable	Independent variable	Standardized regression coefficient(β)	***p***-value	R^**2**^	R^**2**^ adj	ΔR^**2**^ adj	ΔF	***p***-value	VIF	AIC	BIC
50% condition Lumbar-pelvic MARP	Step 1:		0.92	0.01	0.01	0.01	0.08	0.92		211.15	216.88
	Age	0.40						0.83	1.01		
	Duration	0.07						0.73	1.01		
	Step 2:		0.14	0.31	0.17	0.16	2.67	0.06		207.72	219.19
	Age	0.27						0.19	1.35		
	Duration	0.17						0.37	1.13		
	Pain NRS	−0.12						0.54	1.22		
	TSK-11	−0.64						< 0.01	1.63		
	PCS-4	0.30						0.14	1.38		
	FreBAQ	0.06						0.79	1.38		
	Step 3:		< 0.01	0.56	0.43	0.26	12.85	< 0.01		195.96	208.87
	Age	0.18						0.29	1.38		
	Duration	0.13						0.40	1.13		
	Pain NRS	−0.12						0.45	1.22		
	TSK-11	−0.27						0.20	2.18		
	PCS-4	0.26						0.13	1.38		
	FreBAQ	0.10						0.54	1.39		
	Task-specific fear	−0.61						< 0.01	1.52		

*FreBAQ* Fremantle Back Awareness Questionnaire, *NRS* Numerical rating scale, *PCS-4* Pain Catastrophizing Scale-4, *TSK-11* Tampa Scale for Kinesiophobia-11

## Discussion

We sought to identify motor coordination patterns by the measurement of in-phase upper-lower lumbar movement during the lifting of an object, and we investigated how pain-related fear is related to the trunk coordination pattern in workers with CLBP. The results of our analyses demonstrated that the MARP during trunk extension movement in the 50% condition was significantly decreased in the subjects with CLBP compared to the healthy controls. Further, the hierarchical multiple regression analysis revealed that a decreased MARP that was uniquely associated with task-specific fear was evoked when the subjects performed the lifting task.

A lower MARP indicates a more in-phase trunk coordination pattern [13]. Individuals with CLBP have demonstrated a more in-phase trunk coordination pattern during the performance of various tasks compared to individuals without back pain [32, 38]. Such a coordination pattern may be a compensatory behavior resulting from trunk sensorimotor control disorders [13]. Some researchers suggested that sensorimotor control disorders in individuals with CLBP are characterized by altered trunk muscle activation [39] and modulated trunk inner and outer muscle coordination [40], and that the disorders are associated with decreased trunk stability. The mechanical load during a lifting task increases in proportion to the weight of an object [41], and thus a more in-phase coordination pattern might be an adaptive behavior for increased trunk stability.

The results of the present hierarchical multiple regression analysis revealed that the subjects who felt greater task-specific fear had a more in-phase coordination pattern. This supports our hypothesis that the in-phase coordination pattern was the result of not only the mechanical load but also pain-related fear. In other words, the in-phase trunk coordination pattern can be interpreted as one of the avoidance behaviors that are due to pain-related fear. Fear of movement and movement behavior have been shown to be related, among them, Matheve et al. stated that the TSK (i.e., general fear) was inadequate for measuring the association between fear of movement and impaired trunk movement, and they recommended the use of a series of photographs of daily and labor activities (e.g., lifting, shoveling soil) to assess movement behavior [34]. They did not assess task-specific fear, but our present observation that subjects’ task-specific fear should be assessed instead of using the TSK is consistent with their study [34]. We performed a retrospective assessment of our subjects’ task-specific fear because people with CLBP tend to overestimate fear during actual behavior, corrective experience diminishes expected fear. In fact, a previous study assumed that such corrective experience diminishes expected fear [42]. Therefore, a retrospective assessment might be more predictive of movement behavior by correcting for errors between predicted and actual fear. However, the retrospective assessment may be influenced by the previous experience of performing the task itself, and this may potentially have inflated the relationships between these two.

On the other hand, we detected no significant correlation between the kinematic factors (i.e., the MARP and the DP) and other pain-related factors. This is inconsistent with reports that the TSK-11 and PCS-4 scores were significantly related to impaired trunk movement [7, 43]. The TSK and PCS are important assessments to determine general pain-related negative emotion, which might be useful when a relationship between movement and pain is clear, such as in acute LBP and severe CLBP. Those studies [7, 43] examined LBP patients at medical facilities and subjects with CLBP who had received a diagnosis and recommendation for conservation treatment from an attending physician. In contrast, in this study, we examined workers with chronic LBP who were able to continue to work despite experiencing LBP. This type of work-related LBP is characterized by a mild degree of pain intensity and disability [44]. We also compared these assessments with those obtained in previous studies: the TSK-11 score in the present CLBP group was 21.9 ± 5.0 (vs. 27.7 ± 2.4 [7]), and their PCS-4 score was 6.4 ± 3.9 (vs. 8.8 ± 0.9 [44]), demonstrating lower scores in the present CLBP population. Differences in the pathogenesis of LBP might thus have affected the relationship between kinematic factors and pain-related factors.

We also observed no significant differences in the DP between our CLBP and HC groups. Individuals with CLBP have been reported to have less upper-lower lumbar coordination variability during walking compared to healthy individuals [45]. The kinematic characteristics of lifting an object may have affected this result in the present study. Comparing to walking, a lifting task requires a much larger trunk range of motion [12, 46], and thus greater kinematic variance can be generated during a lifting task; as a result, the DP was not influenced by the presence of CLBP.

Our study has some limitations to consider. (1) Because the sample size was small (CLBP, *n* = 31; HC, *n* = 20), and recruitment of participants were from single facilities, the results should be interpreted with caution and their generalization remains unclear. The power was slightly low at 0.70. In addition, the difference in sample size between the two groups might have affected the results. This study should be regarded as a preliminary investigation, and further investigations with large sample sizes are necessary to confirm our findings. (2) We used only a kinematic analysis as a measure of movement behavior; we did not perform kinetic or electromyographic analyses. Investigating other variables such as muscle activity patterns and the lower-back load may help clarify the relationship between motor behavior and pain-related fear. (3) Our subjects were only male healthcare workers, limiting the generalizability of our findings. (4) This was a cross-sectional study, and it was not possible to design an intervention based on a longitudinal course. The development of an intervention based on a longitudinal period is necessary to determine the effects of pain-related fear on kinematic performance. (5) We measured fear based on subjective self-reporting rather than physiological measures (e.g., skin conductance). The subjective self-reports were collected retrospectively, whereas the physiological measures are collected during the task. Experiments that clarify causal relationships between fear and trunk coordination patterns by measuring physiological measures might be needed. (6) The subjects’ self-reports about fear were obtained as a retrospective assessment (after the task performance), but the validity of this type of assessment has not been established. Prospective assessment (before a task performance) has been described [33, 42].

## Conclusion

We attempted to identify motor coordination patterns of the in-phase upper-lower lumbar regions during the lifting of an object and to determine how pain-related fear is related to the trunk coordination pattern in workers with CLBP. The results demonstrated that (1) the upper-lower lumbar motion coordination pattern in the CLBP group was a more in-phase coordination pattern associated with the increased physical load, and (2) task-specific pain-related fear influences the in-phase motion pattern. An intervention for task-specific fear may be necessary to improve individuals’ impaired trunk motor coordination.

## Data Availability

The datasets used and/or analyzed during the current study are available from the corresponding author on reasonable request.
